# Structure and Dynamics of Interphase Chromosomes

**DOI:** 10.1371/journal.pcbi.1000153

**Published:** 2008-08-22

**Authors:** Angelo Rosa, Ralf Everaers

**Affiliations:** 1 Max-Planck-Institut für Physik Komplexer Systeme, Dresden, Germany; 2 Institute for Biocomputation and Physics of Complex Systems (BIFI), Zaragoza, Spain; 3 Université de Lyon, Laboratoire de Physique, École Normale Supérieure de Lyon, CNRS UMR 5672, Lyon, France; Fred Hutchinson Cancer Research Center, United States of America

## Abstract

During interphase chromosomes decondense, but fluorescent in situ hybridization experiments reveal the existence of distinct territories occupied by individual chromosomes inside the nuclei of most eukaryotic cells. We use computer simulations to show that the existence and stability of territories is a kinetic effect that can be explained without invoking an underlying nuclear scaffold or protein-mediated interactions between DNA sequences. In particular, we show that the experimentally observed territory shapes and spatial distances between marked chromosome sites for human, *Drosophila*, and budding yeast chromosomes can be reproduced by a parameter-free minimal model of decondensing chromosomes. Our results suggest that the observed interphase structure and dynamics are due to generic polymer effects: confined Brownian motion conserving the local topological state of long chain molecules and segregation of mutually unentangled chains due to topological constraints.

## Author Summary

Eukaryotic genomes are organized in sets of chromosomes. Each chromosome consists of a single continuous DNA double-helix and associated proteins that organize locally in the form of a *chromatin* fiber. During cell division (mitosis) chromosomes adopt a compact form that is suitable for transport. During periods of normal cell activity (interphase), they decondense inside the cell nucleus. Being long-chain molecules (in the case of human chromosomes the contour length of the chromatin fiber is on the order of 1 mm), the random thermal motion of interphase chromatin fibers is hindered by entanglements, similar to those restricting the manipulation of a knotted ball of wool. We have studied the consequences of this effect using computer simulations. Most importantly, we find that entanglement effects cause sufficiently long chromosomes to remain segregated during interphase and to form “territories.” Our model (1) reproduces currently avaliable experimental results for the existence and shape of territories as well as for the internal chromosome structure and dynamics in interphase nuclei and (2) explains why entanglement effects do *not* interfere with the reverse process of chromosome condensation at the end of interphase.

## Introduction

Eukaryotic genomes are organized in sets of chromosomes which are made up by a single continuous piece of DNA and associated proteins [1]. During cell division (mitosis) chromosomes adopt a compact form which is suitable for transport and which can be discerned in a light microscope. During periods of normal cell activity (interphase), chromosomes decondense. More than 100 years ago, Rabl discovered that interphase chromosomes in newt and *Drosophila* remain organized in distinct territories [2]. During the last twenty years similar territories of various shapes have been observed in many organisms [3], a notable exception being budding yeast whose chromosomes appear to mix freely [4],[5].

The function of these territories, the mechanism responsible for their formation, and the reasons for the differences between species are still unclear [4],[6]. In this paper we investigate, if the observed interphase structure and dynamics are the consequence of a generic polymer effect, the preservation of the local topological state in solutions of entangled chain molecules undergoing Brownian motion. This effect plays an important role for the viscoelastic properties of polymeric systems [7],[8]. In the present context, Sikorav and Jannink [9] assumed that interphase nuclei behave as equilibrated polymer solutions and estimated the disentanglement time *τ*_d_ of condensing metaphase chromosomes as *τ*_d_ = 1.5×10^−7^ (#nucleosomes)^3^ s, where “#nucleosomes” is the total number of nucleosomes in a chromosome. A human chromosome of typical size ≈100 mega-basepairs (Mbp) has ≈500,000 nucleosomes [1], i.e., *τ*_d_≈2×10^10^ s (≈500 years). From this prohibitively high estimate Sikorav and Jannink concluded that the process requires substantial topoisomerase-II (topo-II) activity.

Here we reverse the argument. We suggest that interphase nuclei never equilibrate and behave like semi-dilute solutions of *un*entangled ring polymers which are known to segregate due to topological constraints [10]. Within these territories, individual genomic sites are highly mobile and accessible. However, the structure of interphase and metaphase chromosomes remains largely identical from a topological point of view. Thus, instead of being a problem to be overcome by evolution, slow equilibration of long chromosomes *accelerates* the reverse process of chromosome condensation.

### Experimental Evidence and Polymer Theory

Nowadays, the large-length scale structure of decondensed chromosomes can be experimentally studied using Fluorescence in situ Hybridization (FISH): nucleic acids are chemically modified to incorporate fluorescent probes and specific sequences on single chromosomes can be detected [11]. In particular, it is possible to mark different portions of the genome (chromosome painting) and to determine locations of and spatial distances between targeted sites [11]. Chromosome painting indicates that chromosome territories in human nuclei have an ellipsoidal shape with radii of the order of 1 µm [4]. In contrast and as already discovered by Rabl, the interphase nuclei of organisms like newt or *Drosophila* are organized in elongated territories oriented between two poles of the nucleus [2],[3]. Furthemore, there are also organisms such as budding yeast whose chromosomes appear to mix freely or, at least, considerably less organized [4],[5]. The localization of territories inside the nucleus exhibits regular patterns: gene-rich chromosomes in human lymphocytes preferably locate in the nuclear interior while gene-poor chromosomes are typically found closer to the periphery [12],[13]; in contrast, in human fibroblasts positioning of territories was shown to correlate with chromosome size and not with its gene content [14]. In general, interactions between specific chromosome regions and structural elements within the nuclear envelope, such as nuclear pores or nuclear lamina, are believed to shape chromatin organization [15].

**Figure 1:**
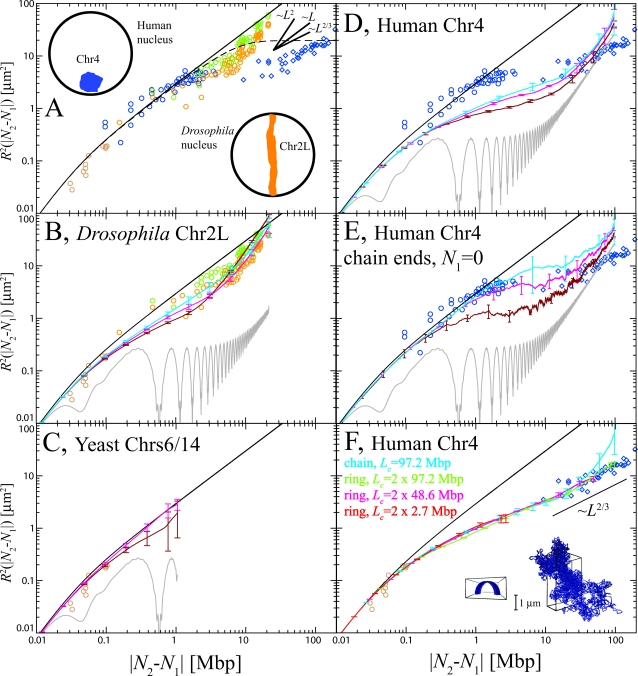
Experimental FISH data for spatial distances *R* ^2^(|*N*_2_−*N*_1_|) between targeted chromosome sites compared to the estimates based on the WLC model (A) and results from our simulations (B–F). *Brown ○*: *Saccharomyces cerevisiae* Chr6 and Chr14 [16]. *Blue ○ and ◊*: *Homo Sapiens* Chr4, |*N*_2_−*N*_1_|<4.5 Mbp and |*N*_2_−*N*_1_|>4.5 Mbp, respectively [17]. *Orange and green ○*: *Drosophila melanogaster* Chr2L, DS5 and DS1 embryos respectively [18]. DS5 and DS1 are two phases of cell cycle. DS1 appears later. *Black continuous line*: Mean-square internal distances predicted by the WLC model, Equation 1. (A) *Black dashed line*: Mean-square internal distances of an ideal polymer chain inside a spherical nucleus of 5 µm radius [7]. (The exact probability distribution function of the square internal distances *R* ^2^(|*N*_2_−*N*_1_|) of a polymer without self-interactions obeys diffusion equation [7] with null boundary conditions (in our case the boundary is the sphere which models the nucleus).)While data for Chr4 and Chr2L show a reasonable agreement at short-length scales, the apparent large-length scale Chr4 behavior *L* ^2/3^ [29] contrasts with the observed *L* ^2^ for Chr2L. The insets show two schematic drawings of the Chr4 territory in a human nucleus (blue) and of Chr2L in Rabl phase in a *Drosophila* nucleus (orange). (B–E) Gray lines represent internal distances in the initial, “metaphase-like” chromosome configuration (Materials and Methods). Internal distances in simulated chromosomes have been averaged over 3 time windows of exponentially growing size: 240 s<*t*<2,400 s (dark red line), 2,400 s<*t*<24,000 s (magenta line) and 24,000 s<*t*<240,000 s (cyan line). Since yeast chromosomes rapidly equilibrate only averages over the first 24,000 s are here reported (panel C). In panel E, *N*_1_ = 0, i.e., has been fixed at the origin of the chain to make equilibration of the chain ends evident. (F) Data from simulations of three ring polymers of decreasing half-size *L*_c_ = 97.2, 48.6, and 2.7 Mbp (green, magenta and red lines respectively). Mean distances seem to extrapolate to an effective power law ∼*L* ^2/3^. *Inset*: Initial (left) and final (right) conformation of a (randomly chosen) half of the largest (2×97.2 Mbp) simulated ring chromosome.

Data on the (relative) position and motion of target sites provide further insight into the organization of interphase chromosomes. In Figure 1A we show average spatial distances between targeted sites as a function of their genomic separation. The figure contains FISH data for yeast chromosomes 6 and 14 (Chr6 and Chr14, brown ○) [16], human chromosome 4 (Chr4, blue ○ and ◊) [17] and *Drosophila* chromosome 2L (Chr2L, orange and green ○) [18]. In the latter case, orange symbols refer to embryos in DS5 phase and green symbols to the DS1 phase which appears later in the cell cycle [19]. Two-dimensional spatial distances between sites on Chr4 measured in fibroblasts cells fixed on microscope slides [17] were here rescaled by 3/2 to obtain the corresponding 3 d distances.

Observations for the various organisms agree on short length scales and coincide with the known properties of the (30 nm) chromatin fiber [16]. Given further the rather structureless appearance of interphase nuclei in the light microscope, a useful starting point for a theoretical description is a confined, equilibrated semi-dilute solution of “worm-like” chromatin fibers [12]. Within the worm-like chain (WLC) model, the fiber statistics can be calculated analytically [7]. It is characterized by a crossover from rigid rod to random coil behavior at a characteristic length scale, the Kuhn length *l*_K_≈300 nm of the 30 nm chromatin fiber [16],[20]. Consider two points located at *N*_1_ and *N*_2_ Mbp from one chosen end of the fiber. They are separated by *L* = |*N*_2_−*N*_1_|×10 µm Mbp^−1^ along the contour of the chromatin fiber [16]. The fiber is essentially stiff with a mean square spatial distance *R* ^2^(*L*≫*l*_K_) = *L* ^2^ on small scales and bent by thermal fluctuations on large scales with *R* ^2^(*L*) = *l*_K_ *L*. The full crossover is described by [21]

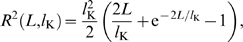
(1)(black continuous line, Figure 1). In particular, Equation 1 holds in the bulk of semi-dilute solutions where chains strongly overlap. Given the typical contour length of *L*_c_ = 1 mm of the chromatin fiber of a human chromosome with ≈100 Mbp, the expected chain extension of 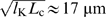 largely exceeds the nuclear radius of 5 µm. In an equilibrated solution, the fibers should fill the nucleus homogeneously with mean-square internal distances saturating at a limiting value (black dashed line, Figure 1A). (The exact probability distribution function of the square internal distances *R* ^2^(|*N*_2_−*N*_1_|) of a polymer without self-interactions obeys diffusion equation [7] with null boundary conditions (in our case the boundary is the sphere which models the nucleus).) In contrast, the smaller yeast (*S. cerevisiae*) chromosome (≈1 Mbp, *L*_c_≈0.01 mm, 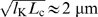) should be only weakly affected by a confinement to its nucleus of ≈1 µm radius [22] while *Drosophila* chromosomes (≈20 Mbp, *L*_c_≈0.2 mm, 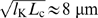) in embryonic cells (for which FISH data are avalaible [18]) are confined inside nuclei whose radius grows from ≈2 µm to ≈5 µm in ≈30 minutes. Not surprisingly, the large-scale statistics of human and *Drosophila* chromosomes does not agree at all with the predictions of a WLC model assuming confinement at the scale of the nucleus (Figure 1A). Rather, the data reflect the different territory shapes observed for the two species. Note, however, that confinement on large scales alone cannot explain the unexpectedly small distances on intermediate scales |*N*_2_−*N*_1_|>4 Mbp for Chr4 (blue ◊).

There is less data available for the dynamics of interphase chromosomes. In mammalian cells chromatin domains of ∼1 µm diameter display little or no motion in a period of several hours [23]. Cabal et al. [24] followed the motion of a marked active gene (*GAL*) in in-vivo yeast nuclei. They observed a mean-square displacement (msd) *g*_1_(*t* = 100 s)≈0.1 µm^2^ for their largest observation interval, i.e., much less than the typical territory size in organisms with larger chromosomes. In particular, the authors reported anomalous diffusion with *g*_1_(*t*)∼*t* ^0.4^.

**Figure 2:**
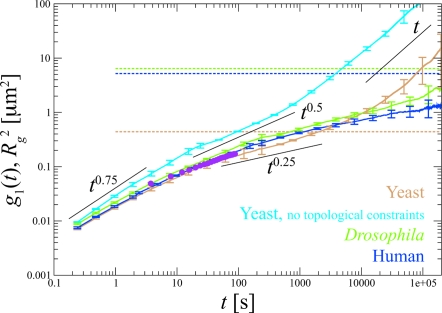
Time behavior of the msd of the six inner beads (*g*_1_(*t*), continuous lines), compared to the average square gyration radius 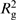 (horizontal dashed lines) of the whole chromosome and measurements of the msd of the active *GAL* gene inside in vivo yeast nuclei [24] (purple dots). For comparison, *g*_1_(*t*) for yeast chromosomes *without* topological constraints has been shown (cyan line). On short time scales, our model reproduces the typical dynamics of semi-flexible polymers with *g*_1_(*t*)∼*t* ^0.75^ [25]. For the model with constraints, there is no extended Rouse regime due to the insufficient separation of Kuhn and entanglement length. Nevertheless, we observe the characteristic *g*_1_(*t*)∼*t* ^0.25^ regime for entangled, flexible polymers [7].

To rationalize this result, it is again useful to consider “worm-like” chromatin fibers in equilibrated semi-dilute solutions at typical nuclear densities. Neglecting entanglement effects, *g*_1_(*t*) displays crossovers between different regimes: (1) *g*_1_(*t*)∼*t* ^0.75^ up to length scales of ≈1 Kuhn length [25]; (2) *g*_1_(*t*)∼*t* ^0.5^ (Rouse behavior) up to length scales of the chain radius of gyration 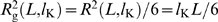 [7]; and (3) *g*_1_(*t*)∼*t* at larger times, when the monomer motion is dominated by the center-of-mass diffusion (cyan line, Figure 2).

In semidilute solutions, linear chains with a contour length exceeding a characteristic value, *L*≫*L*_e_, become mutually entangled, leading to confinement to a tube-like region following the coarse-grained chain contour and a drastically altered, “reptation” dynamics [7]. Estimating *L*_e_ is not a trivial task. How strongly linear polymers entangle with each other depends on their stiffness and on the contour length density of the polymer melt or solution [26]. The latter is most suitably expressed in terms of the density of Kuhn segments, *ρ*_K_. In loosely entangled systems with 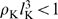 the mean-free chain length between collisions is larger than the Kuhn length, leading to random coil behavior between entanglement points. In contrast, for 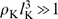 filaments are tightly entangled and exhibit only small bending fluctuations between entanglement points. For a solution of chromatin fibers at a typical nuclear density of ≈0.012 bp/nm^3^ and a Kuhn length of 300 nm (Table 1) 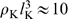, i.e. the system is loosely entangled, but close to the crossover between the limiting cases. The entanglement contour length, *L*_e_, can be estimated via [26] 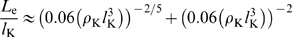, yielding *L*_e_≈1.2 µm or four times the Kuhn length. To a first approximation, chains can thus be considered to be flexible on the tube scale, i.e., we expect around a msd of 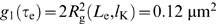 a crossover from Rouse behavior to a *g*_1_(*t*)∼*t* ^0.25^ regime characteristic of reptation [7]. Interestingly, this estimate coincides with the observations of Cabal et al. [24], who reported an intermediate effective power law *g*_1_(*t*)∼*t* ^0.4^ for msd 0.05 µm^2^≤*g*_1_(*t*)≤0.17 µm^2^. Using their data, we can obtain estimates for the entanglement time, *τ*_e_≃32 s, as well as for the disentanglement times, *τ*_d_≈*τ*_e_(*L*_c_/*L*_e_)^3^ [7], of the order of *τ*_d_≃2×10^4^ s, 2×10^8^ s (≈5 years) and 2×10^10^ s (≈500 years) for yeast, *Drosophila* and human chromosomes, respectively. Since this exceeds the life time of the entire organism (not to mention the much shorter cell cycle of most animal cells [1]), *Drosophila* and human chromosomes do not have the time to equilibrate during interphase. (This conclusion does not change, if we take into account entanglement relaxation via topo-II discussed in [9]. At best, this mechanism could completely remove the barrier for chain crossing, thus converting the system to a solution of phantom chains whose relaxation time is given by the Rouse time *τ*_R_≈*τ*_e_(*L*_c_/*L*_e_)^2^ [7]. Yeast, *Drosophila* and human chromosomes would relax in, respectively, 2×10^3^ s, 10^6^ s (≈10 days), and 2×10^7^ s (≈250 days).)

**Table 1:** Summary of the relevant physical parameters for the polymer model of interphase chromosomes.

Parameter	Value
Typical nuclear radius of a human cell [20]	5 µm
Radius of the yeast (*S. Cerevisiae*) nucleus [22]	1 µm
Length of the diploid human genome^a^	6×10^9^ bp
Length of the diploid *Drosophila* genome^a^	3×10^8^ bp
Length of the diploid yeast (*S. Cerevisiae*) genome^a^	2×10^7^ bp
Compaction ratio of chromatin [16]	10^2^ bp/nm
Kuhn length of chromatin [16], *l*_K_	300 nm
Volume fraction of chromatin	10%

a See, e.g., the website http://www.ensembl.org/index.html.

While the discussion up to this point has shed some light on various aspects of the structure and dynamics of interphase chromosomes, we have so far evaded the central question, the origin of the observed chromosome territories. A priori, segregation or (micro) phase separation due to small chemical differences between chains is a common phenomenon in polymeric systems [21]. Organisms could, in principle, render different chromosomes immiscible by a labeling technique akin to chromosome painting. In practice, it is difficult to conceive a corresponding, self-organizing molecular mechanism. Here we propose that the formation of chromosome territories could be related to a different, less well-known effect, the segregation of *unentangled* ring polymers in concentrated solutions due to topological barriers [10],[27]. Well-separated metaphase chromosomes are clearly unentangled at the onset of interphase. Initially, decondensing chains can only rearrange locally and spread out uniformly without changing the global topological state. Up to the extremely long relaxation times for large chromosomes, interphase nuclei should therefore show a behavior similar to concentrated solutions of unentangled ring polymers. In particular, the chromosomes should remain segregated!

It is instructive to compare this explanation to previously published models describing interphase chromosomes as *equilibrium* structures. The unexpectedly small distances on intermediate scales |*N*_2_−*N*_1_|>4 Mbp for Chr4 (blue ◊) were rationalized in terms of giant loops of fibers departing from an underlying (protein) backbone [17] or alternatively, in terms of random loops on all length scales resulting from specific chromatin-chromatin interactions [28]. Simulations of a multi-loop subcompartment polymer model reproduced the experimental observations on human Chr4, by *imposing* (and hence not explaining) confinement to a spherical territory [20],[29]. We do not exclude the possibility of such contacts. However, we claim that territories should also form, if the involved proteins are disabled. For the inverse test—to keep the linking proteins, but to equilibrate a nucleus with disabled local topology conservation—it would be instructive to investigate the structure of nuclei in long-living cells arrested in interphase and to devise ways to maximize the efficiency of topo-II. (This conclusion does not change, if we take into account entanglement relaxation via topo-II discussed in [9]. At best, this mechanism could completely remove the barrier for chain crossing, thus converting the system to a solution of phantom chains whose relaxation time is given by the Rouse time *τ*_R_≈*τ*_e_(*L*_c_/*L*_e_)^2^ [7]. Yeast, *Drosophila* and human chromosomes would relax in, respectively, 2×10^3^ s, 10^6^ s (≈10 days), and 2×10^7^ s (≈250 days).) We note that a few cross-links or attachment points to a residual skeleton would be sufficient to suppress chromosome equilibration via reptation [30]. Long-lived contacts could thus stabilize the observed structures without being at their origin.

How much of the experimental observations can be explained by this topology-conserving, parameter-*free*, minimal model of decondensing chromosomes? Unfortunately, it is difficult to derive quantitative predictions from an analytical theory due to the non-trivial initial conformation, the simultaneous presence of various crossovers (stiff/flexible, loosely/tightly entangled), and the lack of a theory describing the conformational statistics and dynamics of the unentangled ring polymer melts. We have therefore resorted to Molecular Dynamics (MD) computer simulations as a tool which allows us to study the model without further approximations.

### The Model

With a spatial discretization of 30 nm (corresponding to the bead diameter), the employed generic bead-spring polymer model [31] accounts for the linear connectivity, self-avoidance and bending stiffness of the chromatin fiber (Materials and Methods). In particular, there is an energy barrier of 70*K*_B_ *T* to prevent chain crossing [32]. We emphasize that our description does not invoke any protein-like machinery as the nuclear matrix [33]. Furthermore, we neglect local changes of the chromatin fiber as they occur, e.g., as a result of chromatin remodeling during transcription [34], because these processes do not alter the local topological state of the fiber and are therefore irrelevant for the phenomenon we discuss. This argument does not hold for the action of topo-II whose role is precisely to (dis)entangle DNA allowing strands to cross [9],[20]. Non-directed topology changes with a particular rate could be included by suitable modifications of the energy barrier for chain crossing [35]. Similarly, it is straightforward to include (protein-mediated) interactions between specific DNA sites or effects such as confinement by or anchoring to the nuclear envelope [11],[33],[36]. However, here we concentrate on the generic case of decondensing long, internally and mutually unentangled polymers at total concentrations far above the overlap concentration.

**Figure 3:**
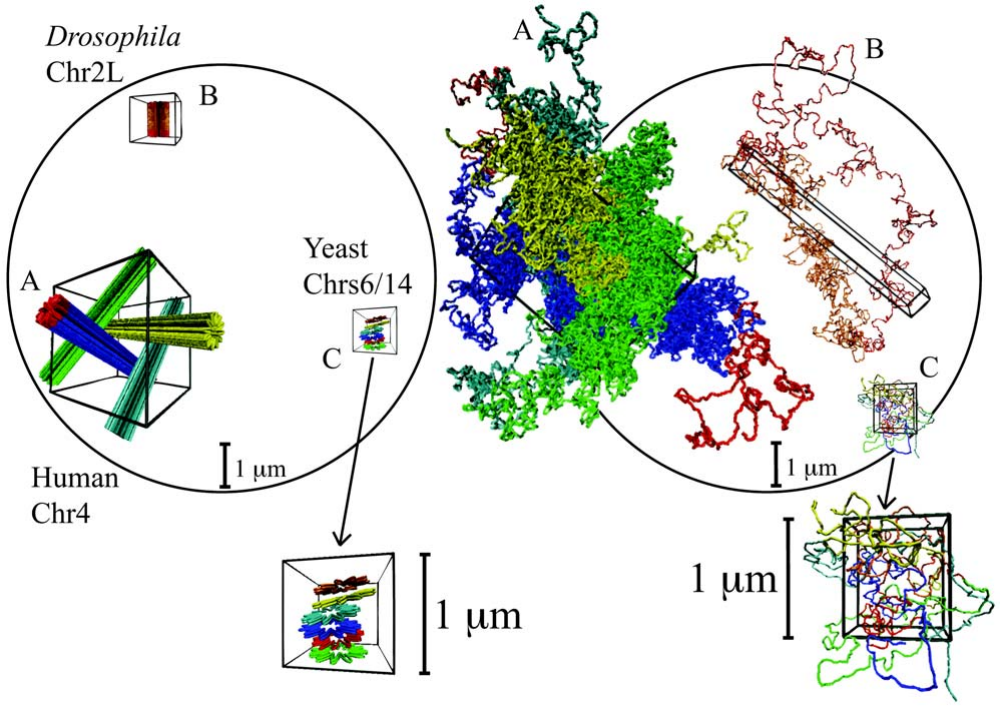
Initial (“metaphase-like”, left) and final (right) configurations of human Chr4 (A), of *Drosophila* Chr2L (B) and of yeast Chr6 and Chr14 (C) shown together with the spherical nucleus (black circle) of 10 µm in diameter and the corresponding simulation boxes (in black). For the blue configuration in A and for the configuration B, we have highlighted in red the two terminal parts up to 4.5 Mbp. In Chr4, this corresponds to the terminal 4p16.3 region [17]. (A) Simultaneous decondensation of 4 model chromosomes half the size the human Chr4. (B) Decondensation of 1 model chromosome the size the *Drosophila* Chr2L. The final elongated shape qualitatively resembles a Rabl-like territory. (C) Simultaneous decondensation of 6 model chromosomes the size the yeast Chr6 and Chr14. Arrows points at magnified versions of the same configurations. Lack of chromosome territoriality is evident.

As initial states of our simulations we chose linear or ring-shaped helical structures remnant of metaphase chromosomes (Materials and Methods). Given the anisotropic shape of our “metaphase” chromosomes, we were interested to see how the decondensation is affected by the presence of other chains. The l.h.s. panel in Figure 3 shows the initial chromosome conformations in our simulations on a common scale, indicated by a typical human nuclear radius of 5 µm. For *Drosophila* (marked “B”, only one chromosome is shown for clarity) we assumed that 8 Chr2L model chromosomes are initially aligned along the common axis of a rectangular simulation box (*nematic* orientation). In the case of yeast (marked “C”) and of the human (marked “A”), we followed the decondensation of 6 respectively 4 chromosomes of equal size which were oriented randomly in the simulation box [14]. For comparison we have also studied ring shaped chromosomes (see inset of Fig. 1F) of different length under conditions corresponding to those of the human cell nucleus. 27 small rings (*L*_c_ = 2×2.7 Mbp) were randomly oriented inside the simulation box, while for larger rings (*L*_c_ = 2×48.6 Mbp and *L*_c_ = 2×97.2 Mbp) we limited ourselves to simulations of single chains in contact with their periodic copies in adjacent simulation cells. The setup as a ring allows us to eliminate chain end effects which otherwise play an important role.

**Figure 4:**
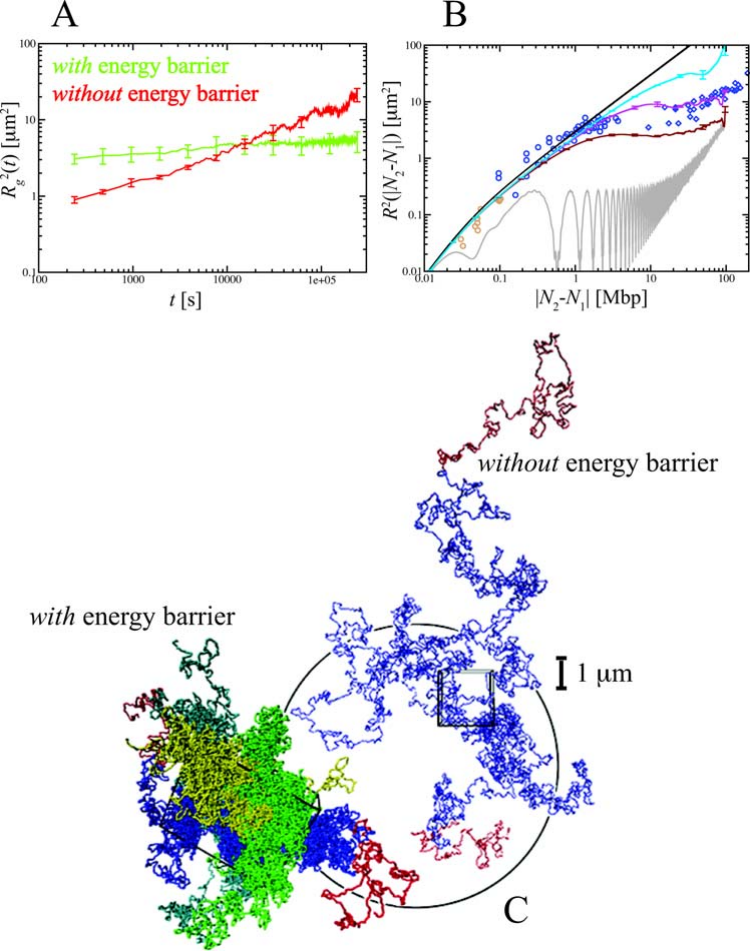
Human Chr4 territories are less stable if the energy barrier against chain crossing is switched off. The swelling from the initial “metaphase” configuration is monitored through the time behavior of the gyration radius 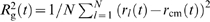 [7], where *r_l_*(*t*) is the position vector of the *l*th bead and *r*_cm_(*t*) is the center of mass of the configuration at time *t*. Without barrier, chromosomes swell easier and have larger size (green and red lines, (A)). Comparison amongst internal distances between two sites located at *N*_1_ and *N*_2_ Mbp from one chosen end of the fiber and avalaible experimental data reflects this behavior (B). We have averaged over 3 time windows of exponentially growing size: 240 s<*t*<2,400 s (dark red line), 2,400 s<*t*<24,000 s (magenta line) and 24,000 s<*t*<240,000 s (cyan line). In particular, we notice that the fortuitous agreement of the magenta line with the data is lost due to the fast relaxation to equilibrium. The gray line corresponds to internal distances in the initial configuration. As expected (C), the final configuration of human Chr4 without energy barrier occupies a larger volume and is more random-walk-like than the ones where the energy barrier has been included.

All simulations were performed in a constant isotropic pressure ensemble using rectangular simulation boxes with three independently fluctuating linear dimensions. The imposed pressure leads to the final density corresponding to the experimental value of ≈0.012 bp/nm^3^ for human nuclei or 10% of volume fraction of chromatin (Table 1). This appears a reasonable choice because the experimental density in yeast nuclei is only two times lower (≈0.006 bp/nm^3^, Table 1), while the rapid growing size of *Drosophila* embryos nuclei [19] does not allow for a univocal choice. We emphasize that the employed periodic boundary conditions do *not* introduce confinement to the finite volume of the simulation box. Using properly unfolded coordinates, chains can extend over arbitrarily large distances (see Figure 4 for the example of a MD simulation using a similar model but with a strongly reduced barrier for chain crossing). To give an idea of the computational effort, we consider the example of Chr4, where we simulated four model chromosomes of half of the actual length of Chr4. Each chromosome is modeled as a chain of 32,400 beads with a total contour length of 10^−3^ m or 97.2 Mbp. Using ≈7×10^4^ single-processor CPU-hours on a CRAY XD1 parallel computer, we followed the dynamics over 12×10^6^ MD time steps. The comparison to the measured single-site mobility for yeast [24] in Figure 2 suggests the value of *τ* ≈2×10^−2^ s used throughout the paper. According to this estimate, we followed the chain dynamics over 240,000 s (≈3 days) of real time. However, it is clear that more experimental data are needed to reliably fix the absolute time scale of our simulations.

## Results

Since there are no attractive interactions in our model of the chromatin fiber, the bent and kinked initial state is unstable and unfolds rapidly. The initial rapid expansion stops when chromosomes come into contact with others, including their periodic replicas in adjacent simulation cells. Our simulation time is sufficient to mix and equilibrate the short (1 Mbp) yeast chromosomes (Figure 3). Fast equilibration of yeast chromosomes explains why apparently there is no territorial organization in yeast nuclei [5]. (Most chromosomes in yeast have a size smaller than 1 Mbp, corresponding to a disentanglement time comparable to the time duration of the relative interphase (∼1 hour [37]).) In this case memory of the initial condition is rapidly lost: a simulation where the chains are initially prepared as rods oriented along the same direction produces similar results (data not shown).

The much longer *Drosophila* and human chromosomes remain confined to distinct territories (Figure 3). For the nematically ordered initial state we assumed for *Drosophila*, we observed that decondensation leads to the formation of Rabl-like elongated territories. In contrast, in isotropically arranged copies of the human Chr4, the preferred axial expansion is suppressed and the resulting territory shapes resemble elongated ellipsoids. Our ring chromosomes essentially reproduce the latter behavior. More quantitatively, the shape of the human Chr4 territory is described by the average of the 3 eigenvalues Λ_1_, Λ_2_, and Λ_3_ of the corresponding gyration tensor ([38] and Materials and Methods) and the ratios of the two largest eigenvalues Λ_1_ and Λ_2_ over Λ_3_ are two quantities which could experimentally be tested. We have found that averaging over the configurations of all the possible sections of half the total ring size gives Λ_1_∶Λ_2_∶Λ_3_ = 6.4(±1.4)∶1.9(±0.4)∶1.0 (2×97.2 Mbp), Λ_1_∶Λ_2_∶Λ_3_ = 5.5(±1.2)∶2.1(±0.5)∶1.0 (2×48.6 Mbp) and Λ_1_∶Λ_2_∶Λ_3_ = 6.9(±0.7)∶2.2(±0.2)∶1.0 (2×2.7 Mbp), while averaging over all the 4-Chr4 configurations gives Λ_1_∶Λ_2_∶Λ_3_ = 11.0(±1.2)∶1.5(±0.3)∶1.0. The ∼2 times larger value found in the latter case is probably an artifact of the setup (see also below).

In Figure 1 (panels B to E) we compare the simulation results for mean-square spatial distances between marked sites on the chromosomes to the experimental findings shown in Figure 1A and discussed in the introduction. Gray lines represent spatial distances between sites in the initial, compact “metaphase” configuration. To give an impression of the time dependence of the results, we have averaged the *R* ^2^(*N*_2_−*N*_1_|) curves over three exponentially spaced time windows: 240 s<*t*<2,400 s, 2,400 s<*t*<24,000 s, 24,000 s<*t*<240,000 s (dark red, magenta and cyan lines respectively). In panels B–D we show results averaged over the entire length of the simulated *Drosophila* chromosome Chr2L, yeast Chr6 and Chr14 and human Chr4. While the former two are in excellent agreement with the experimental data, this is not the case for our first set of results for the human Chr4. Here simulation and experimental data agree quantitatively only on short length scales. It turns out, that there are different explanations for the deviations on intermediate and on large length scales.

Figure 1F shows the corresponding comparison to our data for ring chromosomes. In this case, the experimentally observed conformational statistics of human Chr4 on large scales is perfectly reproduced. In fact, when we reanalyzed data for the linear chromosome assuming the existence of a “centromere-hinge,” we found nearly perfect agreement with the ring data (not shown). This suggests to interpret the (nearly linear) large scale behavior of our simulation results in Figure 1D as an artifact of the straight initial configuration.

Interestingly, the simulation data follow the experimentally observed effective power law *R* ^2^∼*L* ^2*ν*^ with *ν*≈0.32 [29] already on intermediate scales (*L*>1 Mbp). (We note that the relation between the square of the gyration radius 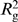 and the mean square internal distances of a polymer *R* ^2^(|*N*_2_−*N*_1_|), 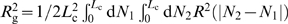 [7], is compatible with chromosome territories being compact objects with 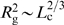. However, the reverse conclusion [20],[29] is incorrect: globular polymer conformations also follow 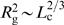, but do not have a fractal structure where the same exponent characterises the entire chain conformation (see, for example the dashed line in Figure 1). Simple confinement alone cannot explain the chain structure.) This behavior seems to be robust, since all our simulation data for linear chains and rings of different size beautifully collapse onto each other. Similar, quasi-fractal structures were reported in [10]. Taken together this suggests that our ring samples are relatively well-equilibrated and that (in agreement with our working hypothesis) long, unentangled linear chains initially relax to a very similar structure. However, we still require an explanation for the deviations between this apparently quite robust prediction and the experimental data in Figure 1D.

**Figure 5:**
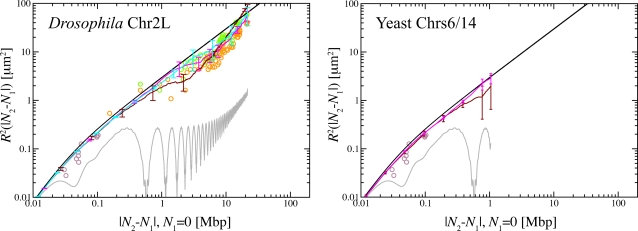
Mean square spatial distances *R* ^2^(|*N*_2_−*N*_1_|) between a site of the fiber located at *N*_2_ Mbp from one chosen end of the chain and the end (here located at *N*_1_ = 0): comparison between simulated and the avalaible experimental data on *Drosophila* Chr2L (left) and yeast Chr6 and Chr14 (right). Gray lines represent internal distances in the initial, “metaphase-like” chromosome configuration (Materials and Methods). Internal distances have been averaged over 3 time windows of exponentially growing size: 240 s<*t*<2,400 s (dark red line), 2,400 s<*t*<24,000 s (magenta line) and 24,000 s<*t*<240,000 s (cyan line). Since yeast chromosomes rapidly equilibrate only averages over the first 24,000 s are here reported. The black continuous line is the plot of the average internal distances predicted by the WLC model, Equation 1.

Reptation theory [30] would suggest that the further equilibration of linear chromosomes proceeds by a very slow escape of the terminal parts of the chain from their initial environment. Qualitatively, this effect is directly observable in Figure 3 where we have marked the terminal parts of our model chromosomes in red. Interestingly, the experimental data for the spatial distances between sites with genomic distances in the Mbp range on human Chr4 were determined in the ≈4.5 Mbp 4p16.3 region which is located at the *end* of *p*-arm [17]. A good way to quantify the consequences is to measure *R* ^2^(|*N*_2_−*N*_1_|,*N*_1_ = 0), i.e., mean-square spatial distances between the chain *ends* and points along the fiber (Figure 1E and Figure 5). These distances show a stronger time dependence than results averaged over the entire chain. In particular, they follow the WLC prediction up to much larger contour length distances before crossing over to the bulk averages. The point of departure from the WLC prediction can be used to estimate up to which distance from the end the chains are equilibrated after a certain time. (The temporal behavior of the ratio between the escaped portion of the chain and the whole contour length *L*_c_ at short times *t* is compatible with the power-law ∼*t* ^1/2^ predicted by reptation theory ([30], data not shown).) The comparison to the experimental data in Figure 1E suggests that the 4p16.3 region of the human Chr4 was nearly equilibrated in the experimental situation. We emphasize that we expect spatial distances between marked sites in the interior of long chromosomes to fall onto the corresponding simulation data in Figure 1D and 1F. This is at least qualitatively supported by a remark in [39] where van den Engh et al. report the more centrally located 6p21 region on human Chr6 to be more compact than the 4p16.3 region near the end of Chr4.

As a final point, we turn to the dynamics of chromosomes during interphase. Figure 2 shows the msd of the 6 inner beads (*g*_1_(*t*)) after the complete (yeast) and initial (human, *Drosophila*) relaxation in comparison to the respective gyration radii 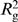. By adjusting the time scale of the simulations, the simulation data can be mapped on the experimental results from [24]. The good agreement indicates that our model provides a simple, quantitative explanation for the reported anomalous diffusion. In particular, the model reproduces the dynamic (entanglement) length scale with no adjustable parameters. Moreover, the dynamic range of the simulation data (0.1 s<*t*<3 days) significantly exceeds the observation window in [24], allowing us to extrapolate to longer times. For comparison, in Figure 2 we have included data for equilibrated yeast chromosome solutions from simulation of a model without excluded volume interactions and topological barriers (cyan line). All simulations exhibit identical short time behavior in agreement with theoretical expectations [25]. A Rouse regime for 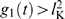 is only observable in simulations without topological barriers. The yeast chromosomes in *equilibrated* entangled solutions exhibit instead *g*_1_(*t*)∼*t* ^0.25^ reptation dynamics. Interestingly, our data for human and *Drosophila* chromosomes show the same behavior in spite of the very different microscopic topological state and the (on these scales) weakly perturbed chain statistics. (The small deviations from the yeast data are artifacts of the constant-pressure simulations used for human and *Drosophila* chromosomes.) In our simulations the asymptotic free diffusion regime—where the center of mass has moved farther than the chain size [7]—is reached only for yeast chromosomes. (Note that the corresponding simulation data cannot be compared directly to experiments, since we have neglected the nuclear confinement in the present study.) While human and *Drosophila* chromosomes remain confined to their territories and do not equilibrate, individual sites are extremely dynamic. Cabal et al. [24] reported that invidual loci on yeast chromosomes explore regions of linear size ∼0.4 µm. The simulations indicate that msd's of ∼1 µm^2^ are reached on the time scale of ∼5 hours.

## Discussion

**Figure 6:**
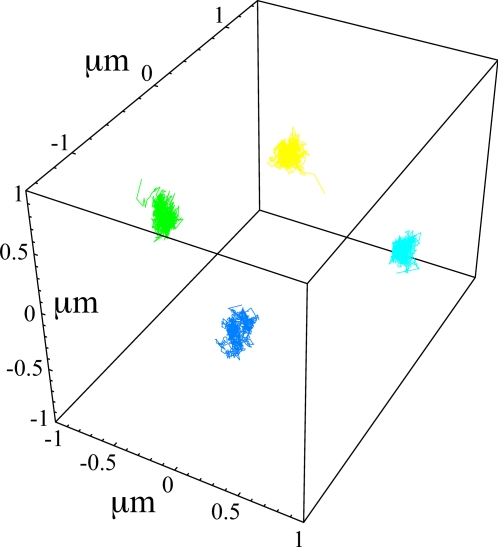
Three dimensional spatial trajectories of the centers of mass of the 4 simulated human Chr4. The color code used corresponds to the snapshots A shown in Figure 2. Motion resembles confined diffusion.

We have studied the decondensation, structure and dynamics of interphase chromosomes using Molecular Dynamics simulations of a bead-spring model of the 30 nm chromatin fiber. Our results suggest that *for sufficiently long* chromosomes territories form as a consequence of a generic polymer effect, the preservation of the local topological state in solutions of long chain molecules undergoing Brownian motion. In fact, we argue that such interphase nuclei never equilibrate and behave like concentrated solutions of unentangled ring polymers, which segregate due to topological constraints [10]. Such cases are also know from material science where they result in unusual material properties [40]. The slow kinetics leads to memory effects. For example, different chromosome arrangements in the nucleus at the end of metaphase provide a rationale for the different territory shapes observed in humans and flies. Similarly, the negligible relative motion of territories provides a natural explanation for the tendency of chromosomes to “reappear” at the end of interphase at similar relative positions as those occupied at the end of the preceeding anaphase [41]. Our simulations confirm this tendency: the centers of mass of the large human chromosomes remain confined to small regions of linear size ≈0.1 µm and retain their relative positions (Figure 6). In contrast, individual sites are extremely dynamic inside the territories and explore much larger regions up to a linear size of ≈1 µm (Figure 2). We emphasize that conservation of the local topology during *decondensation* discussed in the present work considerably simplifies the reverse process of chromosome *condensation* at the end of interphase, a process which takes only about 1 hour in most animal cells [1] and which is difficult to conceive for fully equilibrated nuclei [9].

Obviously, there is more to the structure and dynamics of eukaryotic nuclei than can be captured by the present model in its basic form. However, our results suggest that effects such as active transport [22], chromosome anchoring to the nuclear envelope [36], replication [34] and homologous pairing [19] should be investigated in the framework of the polymer description presented here. As we have shown, computer simulations along the present lines can now reach the relevant time and length scales.

## Materials and Methods

### The Bead-Spring Polymer Model

To model chromatin fiber we used the generic bead-spring polymer model of Kremer and Grest [31]. Chains are composed of interacting beads of diameter *σ*. There are three types of interactions: *U*_LJ_, *U*_FENE_, and *U*_stiff_. *U*_LJ_ is a shifted, purely repulsive Lennard-Jones potential
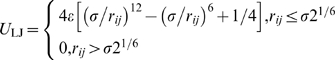
between any two monomers. The potential
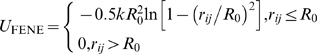
gives the additional interaction between nearest neighbours along the chain. Finally, the stiffness of the fiber is modeled by
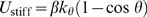
where *θ* is the angle formed by the oriented unit vectors of two consecutive bonds. The bead diameter *σ* equals 30 nm, thus each bead corresponds to 3,000 bp [2]. The other parameters are given by *R*_0_ = 1.5*σ*, *k* = 30.0*ε*/*σ* ^2^, and temperature *K*_B_ *T* = 1.0*ε* [31]. Since the Kuhn's length of the 30-nm fiber is *l*_K_ = 300 nm = 10*σ* [16],[20], the stiffness constant *βk_θ_* is taken = 5 [42].

### Design of the Initial Configuration

Experimental evidence suggests that metaphase chromosomes are folded into loops 30–100 kbp long (rosettes), arranged radially along the axis of the chromatid (see [9] and references therein). Metaphase chromosome are ≈700 nm thick and the length of each chromosome is related to its size [6]. On average, a typical human chromosome has 10^8^ bp, i.e., a contour length *L*_c_ = 10^6^ nm and a length *h*_chr_≈5,000 nm [43].

As a starting configuration, we have placed chain beads along the generalized helix described by the equation:
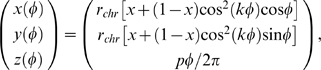
where *r*_chr_ = 12*σ*, *p* = *σ*, and *h*_chr_ = 170*σ*. With this choice of parameters, the length of each turn is approximately = 200*σ*. Given an average loop length of 50 kbp ≈17*σ*, we have ≈12 loops/turn. That fixes the remaining parameters *k* = 6 and *x* = 0.38.

The contour lengths of the *simulated* human Chr4 and *Drosophila* Chr2L are, respectively, *L*_c_ = 97.2 Mbp and *L*_c_ = 21.6 Mbp, which corresponds to chains composed of 32,400 and 7,200 beads.

The ring setup is described by the following equation:
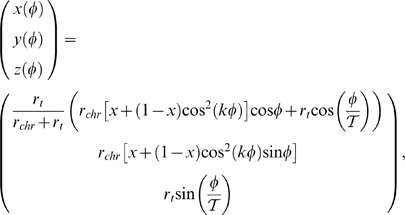
where the period 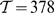, 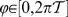, and *r_t_* = 42*σ*.

### Details of the Simulations

The simulations have been performed in a *constant isotropic pressure* ensemble. Since the value of the pressure which must be imposed to the system is not known *a priori*, we have designed the following procedure: the decondensation of a ring chain of contour length *L*_c_ = 5.4 Mbp (1, 800 beads) has been simulated in a *constant volume* ensemble and the average diagonal components of the pressure tensor *P_αβ_* (*α*,*β* = *x*,*y*,*z*) [44] have been calculated. We have found *P_xx_* = *P_yy_* = *P_zz_* = 0.01 and this value has been used throughout the paper. However, simulations of yeast chromosomes *dynamics* have been performed in a *constant volume* ensemble, because in the constant pressure ensemble the small system size leads to large *unphysical* fluctuations of the simulation box. In this constant volume ensemble, simulated yeast chromosomes have been initially arranged in an *equilibrated* configuration.

We have chosen the integration time *t*_int_ = 0.012*τ*, where *τ* = *σ*(*m*/*ε*)^1/2^ is the Lennard-Jones time and *m* is the *mass* of each bead [31]. Each simulation runs up to time 10^9^ *t*_int_ = 12×10^6^ *τ*. Since we have sampled each 10^6^ *t*_int_, each running produces 10^3^ configurations.

Notice that the time behavior of the msd of the 6 inner beads (*g*_1_(*t*)) (Figure 2) has been calculated after shifting to the frame where the center of mass of the *whole* system is at rest. For human and *Drosophila* chromosomes, *g*_1_(*t*) and 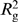 have been calculated neglecting the first 6×10^5^ *τ*≈12,000 s of the simulated trajectory.

### Gyration Tensor

The gyration tensor *T* of an object composed of *N* beads is the 3×3 symmetric matrix whose elements are 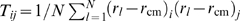, where *r_l_* is the vector pointing at the *l*th bead, 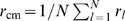 is the center of mass of the beads and *i*,*j* = 1,2,3 are the three indices for cartesian components. The trace of *T*, 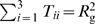 where 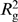 is the square of the gyration radius of the object [7]. It also equals Λ_1_+Λ_2_+Λ_3_, where Λ*_i_* is the *i*th eigenvalue of *T*. For objects with spherical symmetry, Λ_1_ = Λ_2_ = Λ_3_. Then, differences between the eigenvalues measure the anisotropy of the object [38].
